# Low flow rate alters haemostatic parameters in an ex-vivo extracorporeal membrane oxygenation circuit

**DOI:** 10.1186/s40635-019-0264-z

**Published:** 2019-08-20

**Authors:** Katrina K. Ki, Margaret R. Passmore, Chris H. H. Chan, Maximilian V. Malfertheiner, Jonathon P. Fanning, Mahé Bouquet, Jonathan E. Millar, John F. Fraser, Jacky Y. Suen

**Affiliations:** 1 Critical Care Research Group, The Prince Charles Hospital, Brisbane, Australia; 2 Faculty of Medicine, University of Queensland, Brisbane, Australia; 3 Innovative Cardiovascular Engineering and Technology Laboratory, Critical Care Research Group, The Prince Charles Hospital, Brisbane, Australia; 4 Department of Engineering and Built Environment, Griffith University, Gold Coast, Australia; 5 Department of Internal Medicine II, University Medical Center Regensburg, Regensburg, Germany; 6 Wellcome-Wolfson Centre for Experimental Medicine, Queen’s University Belfast, Belfast, UK

**Keywords:** Coagulation, Critical illness, Extracorporeal membrane oxygenation, Flow rate, Haemolysis, Platelets

## Abstract

**Background:**

Extracorporeal membrane oxygenation (ECMO) is a life-saving modality used to manage cardiopulmonary failure refractory to conventional medical and surgical therapies. Despite advances in ECMO equipment, bleeding and thrombosis remain significant complications. While the flow rate for ECMO support is well recognized, less is known about the minimum-rate requirements and haemostasis. We investigated the relationship between different ECMO flow rates, and their effect on haemolysis and coagulation.

**Methods:**

Ten ex-vivo ECMO circuits were tested using donated, < 24-h-old human whole blood, with two flow rates: high-flow at 4 L/min (normal adult cardiac output; *n* = 5) and low-flow at 1.5 L/min (weaning; *n* = 5). Serial blood samples were taken for analysis of haemolysis, von Willebrand factor (vWF) multimers by immunoblotting, rotational thromboelastometry, platelet aggregometry, flow cytometry and routine coagulation laboratory tests.

**Results:**

Low-flow rates increased haemolysis after 2 h (*p* = 0.02), 4 h (*p* = 0.02) and 6 h (*p* = 0.02) and the loss of high-molecular-weight vWF multimers (*p* = 0.01), while reducing ristocetin-induced platelet aggregation (*p* = 0.0002). Additionally, clot formation times were prolonged (*p* = 0.006), with a corresponding decrease in maximum clot firmness (*p* = 0.006).

**Conclusions:**

In an ex-vivo model of ECMO, low-flow rate (1.5 L/min) altered haemostatic parameters compared to high-flow (4 L/min). Observed differences in haemolysis, ristocetin-induced platelet aggregation, high-molecular-weight vWF multimers and clot formation time suggest an increased risk of bleeding complications. Since patients are often on ECMO for protracted periods, extended-duration studies are required to characterise long-term ECMO-induced haemostatic changes.

## Background

Extracorporeal membrane oxygenation (ECMO) is a potentially life-saving modality that is used to provide temporary cardiopulmonary (venoarterial or VA) or pulmonary (venovenous or VV) support for critically ill patients. Despite this modality’s benefits, haemostatic alterations during ECMO are common and involve both bleeding and thrombotic events [1]. Considerable progress has been made in both circuit design and cannulation techniques; however, interaction between blood and the circuit’s artificial surface continues to trigger inflammation and disrupt normal haemostasis [2]. The result is hypercoagulability, counteracted by excessive fibrinolysis and the subsequent consumption of clotting factors, thrombocytopenia and impaired platelet function [3]. To maintain adequate oxygen delivery and CO_2_ removal, a typical ECMO flow rate for an adult is between 60 to 80 mL/kg/min (4 to 6 L/min), which is gradually reduced to less than 30% of that total for weaning [4].

Recommended flow rates for ECMO support have been well-documented [5]; however, less is known about how minimum blood flow rates affect haemolysis and coagulation. This is especially relevant during ECMO weaning. While reducing ECMO blood flow rates is necessary for safe weaning from VA ECMO, weaning practices for VV ECMO show wide variability [6]. The strategies reported by experienced ECMO centres have included (i) maintaining blood flow rates while weaning fresh (or sweep) gas flow; (ii) reducing ECMO blood pump flows while either maintaining fresh gas flows; or (iii) a combination of the two strategies. These differences result from divergent expert opinions and sparsity of evidence regarding the merits of reducing pump flows to lessen shear stress to the blood elements, versus an increased risk of thrombosis. Furthermore, the effect of low-flow also has implications for extracorporeal carbon dioxide removal (ECCO_2_R), where low- and ultra-low-flow (to as low as 250 mL/min) is considered desirable for reducing the invasiveness of extracorporeal supportive therapy in acute respiratory distress syndrome and refractory hypercapnia [7]. Blood flow through ECCO_2_R circuits is achieved either by using the arteriovenous gradient of the patient (pumpless arterio-venous) or using centrifugal or diagonal low-impact flow pumps (veno-venous) [8]. Thus, a more complete understanding of this process will help to optimise both weaning and anti-coagulation management strategies and provide justification for reducing or aiming for low-flow extracorporeal support.

Vascular access cannulae and centrifugal pumps, used for ECMO support, can alter fluid dynamics, often producing high shear stress and turbulent flow [9, 10]. This change in haemodynamics can cause damage to circulating blood cells and haemostatic proteins, and reduce the binding of von Willebrand factor (vWF) to collagen and platelets, resulting in a state of thrombosis, fibrinolysis and compromised platelet function [11]. Enhanced vWF cleavage and the successive loss of its high molecular weight (HMW) multimers is believed to be one of the main causes of bleeding complications in ventricular assist device patients [12]. HMW vWF multimer bands are also lost in patients undergoing ECMO support and acquired von Willebrand syndrome has been linked to the loss of platelet adhesion receptors in these patients [10, 13].

The aim of the current study was to investigate the impact of ECMO when blood circulates at high- (standard practice to match cardiac output) versus low-flow (used while patients are weaned off ECMO). We hypothesised that, relative to low-flow rates, a higher flow rate would increase haemolysis, prolong clot formation times, reduce platelet aggregation and lead to the increased consumption of coagulation factors.

## Materials and methods

### Experimental design and ECMO setup

**Fig. 1:**
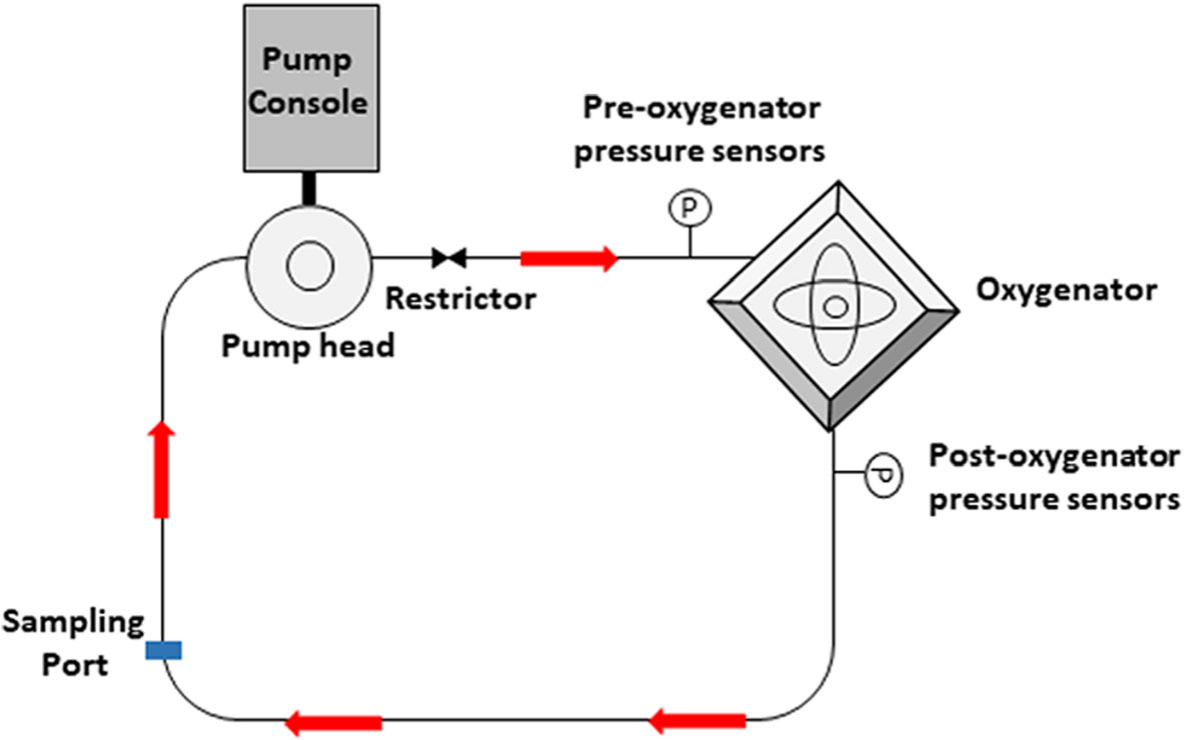
Schematic diagram of ex-vivo ECMO model setup

All methods were approved and performed in accordance with the guidelines and regulations set forth by the Metro North Ethics Committee (HREC/16/QPCH/320). Informed consent for study participation was obtained from the Australian Red Cross Blood Service (17-11QLD-05). Blood circulation loops were developed based upon ASTM F 1841-97 Standard Practice for the Assessment of Hemolysis in Continuous Flow Blood Pumps [14]. Ten ex vivo ECMO circuits (Permanent Life Support System (PLS), Maquet CP, Rastatt, Germany) were tested (Fig. 1). The PLS Set consists of an oxygenator and ROTAFLOW centrifugal pump, both incorporated into a tubing set, with a tip-to-tip BIOLINE (recombinant human albumin and heparin) coating. Donated human whole blood units (Australian Red Cross Blood Service, Kelvin Grove, Queensland) were collected into top and bottom bags with a built in filter, containing CPD (citrate phosphate dextrose; 66.5 mL; Macopharma, Mouvaux, France). Bags were stored at 4 °C and used within 24 h, with blood subsequently circulated at two flow rates: 4 L/min (normal cardiac output; *n* = 5) and 1.5 L/min (weaning; *n* = 5) (total blood volume in the circuit, 420 ± 50 mL). The PLS system was initially primed with 0.9% sodium chloride before whole blood was introduced directly into the circuit from the blood bag. ROTAFLOW centrifugal pump speed was adjusted to 2000 (average 2033 ± 13 rpm) for circulation, and CO_2_ enhanced gas (5% CO_2_, 21% O_2_, 74% N_2_) was added at 2.5 L/min and heat exchange started. Heparin (400 IU; Pfizer, NSW, Australia), calcium chloride (3.33 mg/mL; Phebra, NSW, Australia) and sodium bicarbonate (0.016 mmol/mL; Phebra) were subsequently added. Once flow had stabilized, a vice-grip clamp was used in both high- and low-flow conditions. The vice-grip was tightened to adjust the blood flow rate to 4 L/min for high-flow experiments. For low-flow experiments, the centrifugal pump speed was reduced (average 898 ± 13 rpm) until a flow rate of 1.5 L/min was achieved. Inlet and outlet pressures between the membrane oxygenator were monitored with silicone strain-gauge pressure transducers (PX181B-015C5V; Omega Engineering, CT, USA) and adjusted to 100–150 mmHg using SAGM solution (saline-adenine-glucose-mannitol, MacoPharma, Australia). Calcium (> 2.5 mmol/L), glucose (19.2 ± 4.1 mmol/L) and pH (7.36 ± 0.05) levels were monitored throughout the experiment with rotational thromboelastometry (ROTEM) used to monitor anti-coagulation requirements.

### Blood gas and sample collection

Blood was analysed for electrolytes, glucose and pH using a handheld point-of-care system (Abbott i-STAT, Chicago, ILL, USA). Samples were collected at baseline, 1, 2, 4 and 6 h for full blood examination (FBE), flow cytometry and ROTEM® analysis, as well as for Multiplate® whole blood platelet function testing. After blood sampling, an equivalent volume of SAGM was injected back into the system to maintain pressure at 100–150 mmHg. A full blood examination was determined using the AcT diff™ haematology analyser (Beckman Coulter Australia Pty Ltd, NSW, Australia) to assess the white cell count (WCC), haemoglobin (Hb) level and platelet count (PLT). Samples for vWF multimers and routine/specialized coagulation tests (including ELISA’s and Luminex assays) were centrifuged twice (15 min, 4 °C, 3000×*g*) to obtain platelet-poor plasma (PPP) and subsequently stored at − 80 °C, until further analysis. Samples for haemolysis were centrifuged once (15 min, 4 °C, 3000×*g*).

### Haemolysis assay

Plasma samples (100 μL) were diluted with 1 mL 0.1% Na_2_CO_3_ solution and absorbance measured using a UV/visible spectrophotometer (SmartSpec 3000, Bio-Rad, California, USA) at three wavelengths: 380, 415 and 450 nm. Plasma-free haemoglobin (*pf*Hb) level was calculated using the equation below [15].

$$ \mathrm{pfHb}\ \left(\frac{\mathrm{mg}}{\mathrm{dL}}\right)=\left(167.2\ \mathrm{x}\ A415-83.6\ \mathrm{x}\ A380-83.6\ \mathrm{x}\ A450\right)\ \mathrm{x}\frac{1}{10}\ \mathrm{x}\ \left(1/\frac{\mathrm{Vol}\ \mathrm{plasma}}{\mathrm{Vol}\ {\mathrm{Na}}_2{\mathrm{CO}}_3}\right) $$

### Whole blood platelet function (Multiplate®)

Whole blood platelet aggregometry was measured by change in impedance, using the Multiplate® 5.0—platelet function analyser (Haemoview Diagnostics, Brisbane, Australia). Hirudin-anticoagulated blood was tested using ristocetin (RISTO; 0.77 mg/mL), adenosine diphosphate (ADP; 6.5 μM) and thrombin receptor activating peptide-6 (TRAP-6; 32 μM) agonists. Data was expressed as the area under the aggregation curve (AUC).

### Immunoblotting for high and low molecular weight vWF

Electrophoresis was performed as per previously published methodology [16]. Briefly, samples were run on a gel using high gelling temperature SeaKem agarose (Lonza, Basel, Switzerland) and transferred to a PVDF membrane (Merck KGaA, Darmstadt, Germany). Membranes were blocked using 5% skim milk in TBS/Tween and probed with horseradish peroxidase conjugated polyclonal rabbit anti-human vWF (1:1000) (DAKO, Glostrup, Denmark). Membranes were washed and developed with enhanced chemiluminescence (ECL; Amersham Bioscience, NJ, USA), and band density visualized with the ImageQuant LAS 4000 (GE Healthcare, NJ, USA). Protein expression was quantified using ImageJ (National Institute of Health) and data normalised to baseline measurements.

### Flow cytometry staining

Whole blood collected at baseline, 1 and 6 h was stained with the following monoclonal antibodies (Becton Dickinson (BD) Biosciences, California, USA): fluorescein isothiocyanate (FITC) conjugated CD41a, Brilliant violet (BV)-421 conjugated P-selectin and allophycocyanin (APC) conjugated CD42b (15 min; room temperature). Red blood cells were then lysed (FACS Lysing Solution; BD Biosciences) and samples analysed using a Fortessa X-20 flow cytometer (BD Biosciences). Platelets were first identified based upon forward and side scatter, and further confirmed by gating CD41a positive cells. The P-selectin and CD42b mean fluorescent intensity (MFI) of each sample was analysed using FCS Expression 6 (De Novo Software, California, USA).

### Specialized coagulation tests

The levels of factors XII and XIII, and antithrombin (AT) III were measured in plasma using a standard sandwich ELISA (Abcam, VIC, Australia). Plasma specimens also were analysed using custom Luminex Magpix-based assays (R&D Systems, MN, USA), in accordance with manufacturer instructions. Analytes measured included ADAMTS13, P-selectin, d-dimer and Protein C. Concentrations were calculated using a five-parameter logistic standard curve, corrected for background readings.

### Rotational thromboelastometry (ROTEM®) and routine coagulation tests

Whole blood clot formation profiles were recorded by ROTEM® Thromboelastometry (Haemoview Diagnostics, Brisbane, Australia) with HEPTEM (contact factor-initiated coagulation with heparinase), EXTEM (thromboplastin-initiated coagulation) and FIBTEM (thromboplastin-initiated coagulation with the platelet inhibitor cytochalasin D) activating reagents, in accordance with manufacturer instructions. Parameters evaluated included clotting time (CT), clot formation time (CFT) and maximum clot firmness (MCF). Prothrombin time (PT) was measured on the Stago STAR Evolution analyser (Diagnostica Stago, Doncaster, VIC, Australia), following manufacturer instructions.

### Statistical analysis

Each data set’s distribution was assessed for normality by inspecting the corresponding histogram and pp-plot, and using a Kolmogorov-Smirnov test. Two-way repeated-measures analysis of variance (RM-ANOVA) was used to compare differences between high-flow and low-flow over time for all investigated parameters, except haemolysis and vWF multimers. When simple main effects and/or any interaction between time and condition was significant, a Tukey’s post-hoc test was performed for multiple comparisons. For haemolysis and vWF multimers, the assumption of homogeneity of variances was not met; consequently, Kruskal-Wallis tests were used to compare high-flow or low-flow over time. Measurements between the two experimental groups were compared with Mann-Whitney tests for time point. All analyses considered potential confounders; this included SAGM dilution. All statistical analysis was performed using Statistica, version 13.2 (TIBCO Software Inc, Palo Alto, CA, USA). A *p* value ≤ 0.05 was considered statistically significant.

## Results

### Full blood examination

Haematological parameters were examined at selected time points (Table 1). Baseline measurements did not differ significantly across the experimental groups. WCC, Hb, and PLTs remained constant throughout the experiment, with no significant difference over time or between high- and low-flow time points.

**Table 1:** Haemostatic parameters by experimental group at selected time points

	High flow (4 L/min)				Low flow (1.5 L/min)			
Mean (± SEM)	Baseline	2HR	4HR	6HR	Baseline	2HR	4HR	6HR
Full blood examination								
WCC (× 10^9^/L)	4.7 (0.5)	4.8 (0.5)	4.6 (0.4)	4.8 (0.6)	4.4 (0.4)	4.5 (0.4)	4.4 (0.4)	4.3 (0.4)
Hb (g/L)	98 (2)	101 (2)	99 (3)	104 (3)	102 (6)	103 (6)	103 (6)	106 (7)
Plt (× 10^9^/L)	154 (9)	172 (6)	170 (6)	170 (4)	130 (18)	131 (16)	123 (14)	120 (16)

*n* = 5 high flow, *n* = 4 low flow; *Hb* haemoglobin, *Plt* platelet, *WCC* white cell count

### Haemolysis

**Fig. 2:**
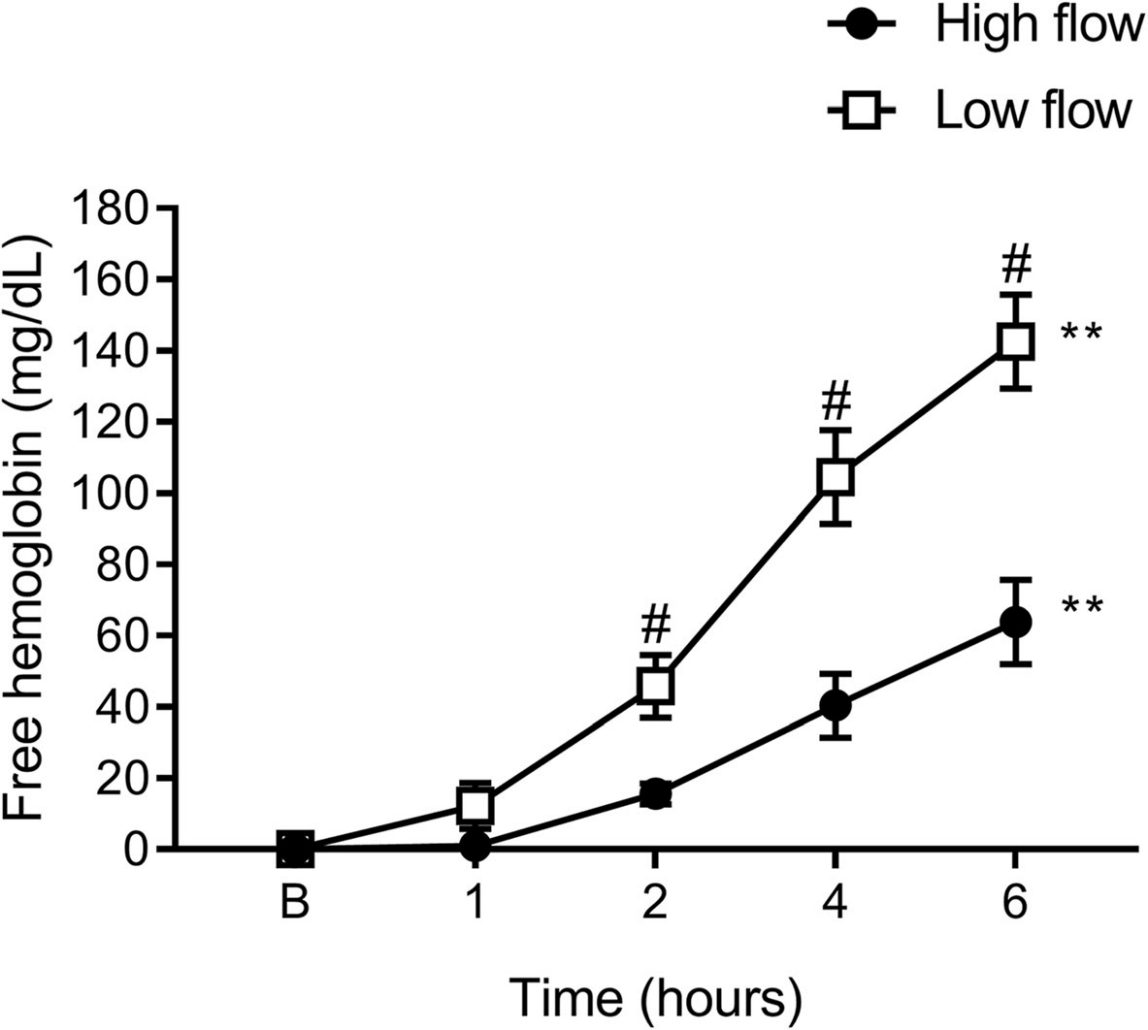
Time-dependent haemolysis with different blood flow regimens. Haemolysis levels were significantly increased over time from baseline to 6 h with both high and low-flow rates. Significant differences also were evident between high- and low-flow at 2, 4 and 6 h. Data are presented as mean ± SEM. **p < 0.01 from baseline to 6 h; #*p* < 0.05 high vs. low flow. *n* = 5 high flow, *n* = 4 low flow

Haemolysis levels were significantly increased over time from baseline to 6 hours, both with high- (*p* = 0.01) and low-flow (*p* = 0.002, Fig. 2). Significant differences between the two flow rates also were evident after 2 h (*p* = 0.02), 4 h (*p* = 0.02) and 6 h (*p* = 0.02) with greater haemolysis during low flow.

### Platelet function (Multiplate®)

**Fig. 3:**
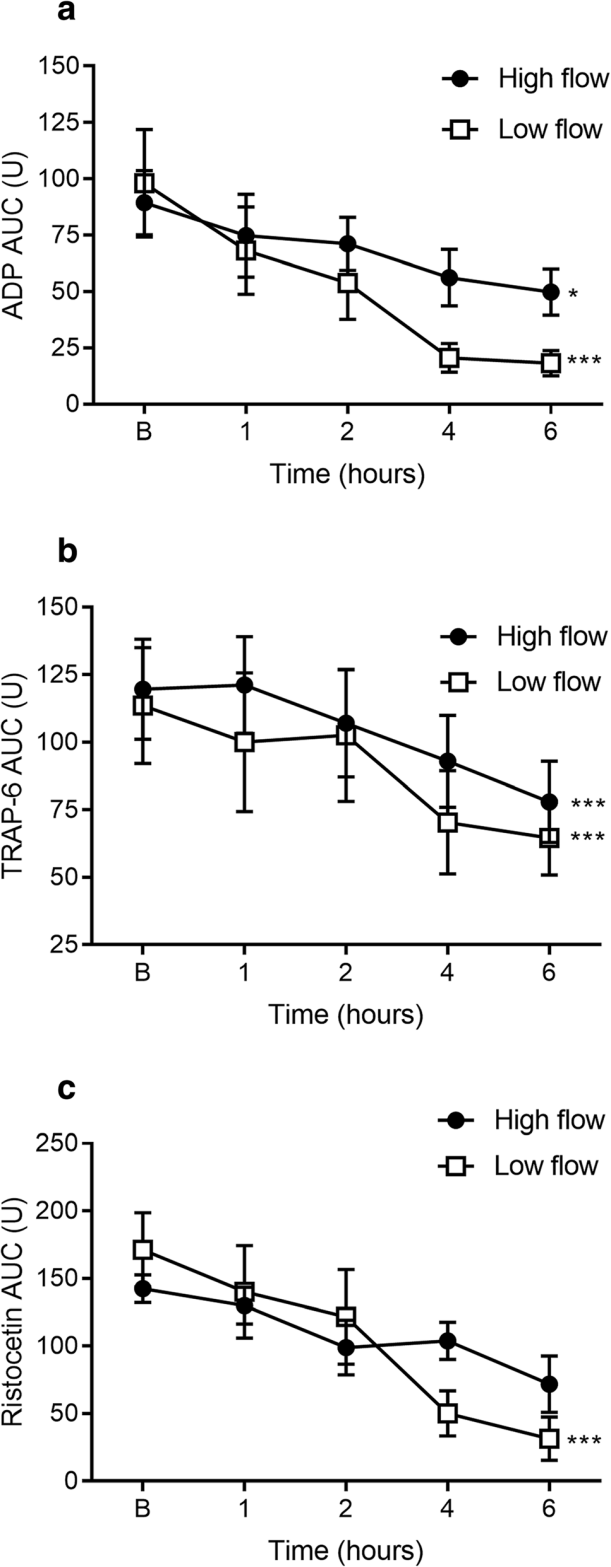
Platelet aggregometry function measured at discrete time points. ADP- (**a**) and TRAP-induced (**b**) platelet aggregation area under the curve (AUC) was significantly decreased over time from baseline to 6 h with both high and low flow. RISTO-induced platelet aggregation (**c**) was significantly decreased over time from baseline to 6 h with low flow only. Data are presented as mean ± SEM. *p < 0.05; ***p < 0.001 from baseline to 6 h. *n* = 5 high flow, *n* = 5 low flow

ADP- and TRAP-induced platelet aggregation were both significantly decreased over time from baseline to 6 h, with high- (*p* = 0.02 and *p* = 0.0008, respectively) and low-flow (both *p* = 0.0002, Fig. 3a, b). RISTO-induced platelet aggregation was significantly decreased over time from baseline to 6 h with low-flow only (*p* = 0.0002, Fig. 3c). There was no significant difference between high- and low-flow at individual time points.

### High molecular weight vWF degradation

**Fig. 4:**
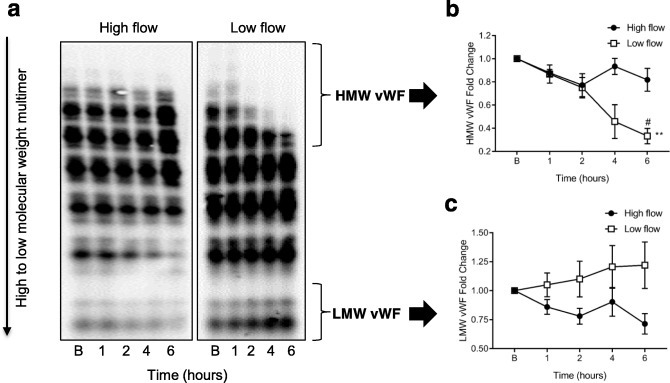
Measurement of von Willebrand factor (vWF) multimers by immunoblotting in whole blood subjected to high and low flow conditions. Representative immunoblot image from two gels run at high flow and low flow (**a**) with loss of high-molecular-weight (HMW) vWF bands (analysed by densitometry). Data normalized to total vWF for HMW multimers (**b**) and LMW multimers (**c**). Data are presented as mean ± SEM. **p < 0.01 from baseline to 6 h; #*p* < 0.05 high vs low flow. *n* = 5 high flow, *n* = 4 low flow

The levels of HMW vWF multimers were significantly reduced over time from baseline to 6 h with low-flow only (*p* = 0.01, Fig. 4). Significant differences between high- and low-flow also were evident after 6 h (*p* = 0.03) with a loss of HMW vWF multimers during low-flow. There was no difference in low molecular weight vWF multimers over time or between the two flow dynamics.

### Flow cytometry and specialized coagulation tests

**Fig. 5:**
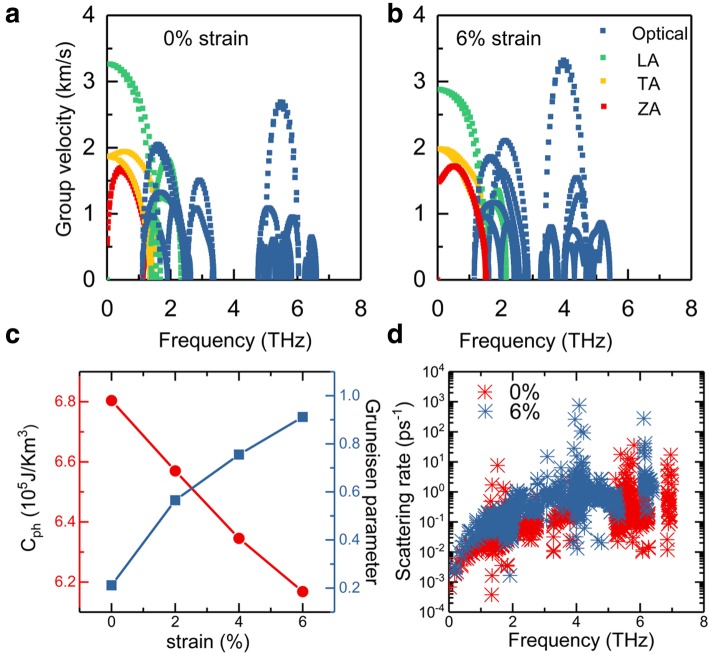
Measurement of specialized coagulation markers. Plasma levels of P-selectin (**a**) were increased over time with both high and low flow. The levels of Protein C (**b**) were increased with high flow, while d-dimer (**c**) levels were increased with low flow. Data are presented as mean ± SEM. *p < 0.05; **p < 0.01; ***p < 0.001 from baseline to 6 h. *n* = 5 high flow, *n* = 5 low flow

No changes were detected in the MFI of P-selectin or CD42b on platelets with either of the different flow rates when assessed by flow cytometry. However, plasma levels of P-selectin were increased from baseline to 6 h with both high- (*p* = 0.0002) and low-flow (*p* = 0.02, Fig. 5a). Protein C plasma levels were significantly increased from baseline at 6 h with high flow (*p* = 0.039, Fig. 5b), while levels of d-dimer were increased at 6 h with low-flow (*p* = 0.003, Fig. 5c). There were no changes in the plasma levels of FXII, FXIII, ATIII, or ADAMTS13 at the different flow rates.

### Thromboelastometry and routine coagulation tests

**Fig. 6:**
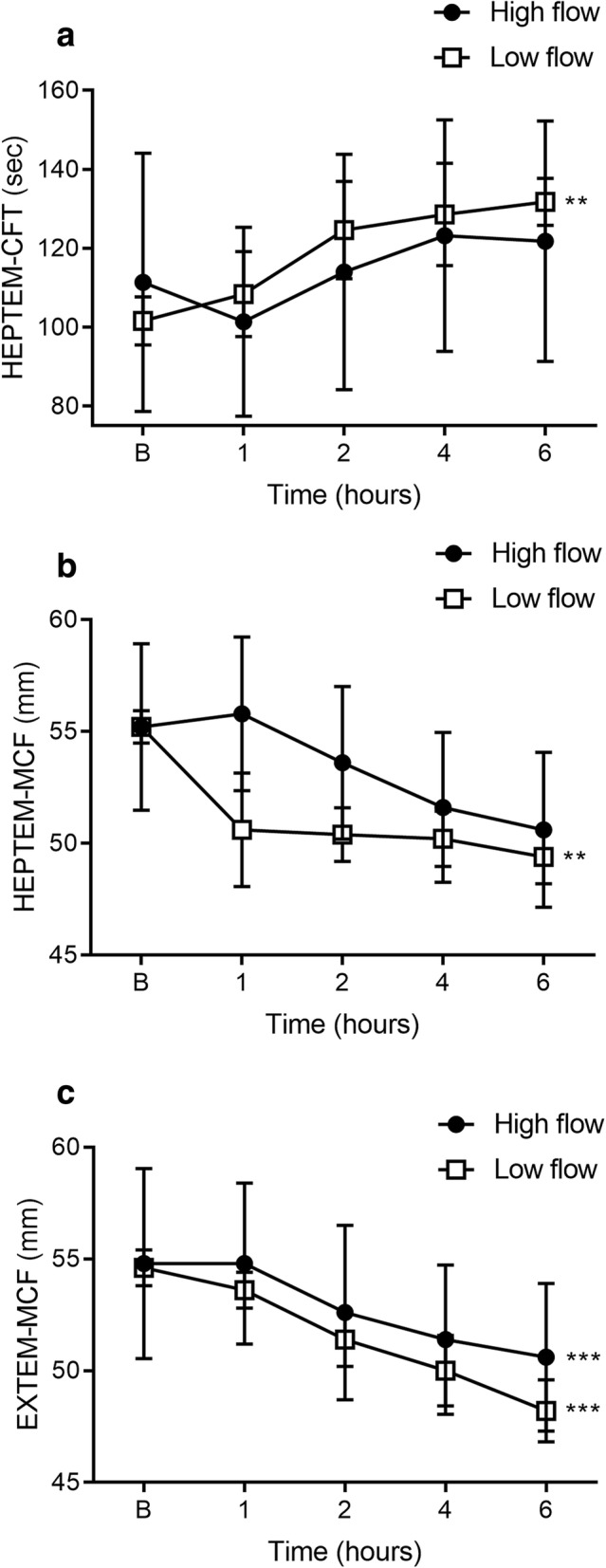
The effect of high vs. low flow on HEPTEM and EXTEM parameters. Low flow resulted in a prolonged HEPTEM-CFT (**a**) with a corresponding decrease in MCF (**b**). EXTEM-MCF was lower with both high and low flow conditions (**c**). Data are presented as mean ± SEM. **p < 0.01; ***p < 0.001 from baseline to 6 h. *n* = 5 high flow, *n* = 5 low flow

Low flow resulted in prolongation of HEPTEM-CFT (*p* = 0.006, Fig. 6a); and overall clot quality, as measured by the HEPTEM-MCF, was lower over time from baseline to 6 h (*p* = 0.006, Fig. 6b). There was no difference between the individual time points during low and high flow. Additionally, EXTEM-MCF was reduced over time from baseline to 6 h with both high (*p* = 0.0002) and low flow (*p* = 0.0002, Fig. 6c). PT and FIBTEM remained unchanged across both flow conditions.

## Discussion

We sought to determine how different flow rates in an ECMO circuit would affect haemolysis and coagulation parameters. Recommended flow rates for ECMO support are well-recognized [5]. On the other hand, lower flow rates and how they alter coagulation have not been thoroughly explored. Typical ECMO flow rates for an adult are between 60 and 80 mL/kg/min (4 to 6 L/min) and are gradually reduced to less than 30% of that total for weaning [4]. However, weaning strategies can differ between ECMO centres [6]. We chose 4 L/min (high-flow) and 1.5 L/min (low-flow) to reflect common practices. Interestingly, we found that a low-flow rate increased haemolysis and the loss of high molecular weight vWF multimers, while it also reduced RISTO-induced platelet aggregation. Clot formation times were also prolonged, with a corresponding decrease in maximum clot firmness.

Haemolysis is a common complication with centrifugal pumps, potentially resulting from a number of factors, including increased shear stress and pressure change across the oxygenator [17]. Increased pump speed is also related to the development of haemolysis [18], as such, our discovery that plasma-free haemoglobin levels are higher with low- compared to high-flow were surprising. Previously published data comparing low-flow rates in ECMO are sparse, with studies in neonates revealing no significant elevation in haemolysis [19, 20]. Cannula size has also been shown to affect shear stress and influence packed red blood cell transfusion [21]. We speculate that haemolysis is influenced by ongoing clotting in the circuit. Computer simulations with ventricular assist devices have identified increased platelet deposition and thrombus growth with flow rates of 2 L/min versus 4.5 L/min [22].

Platelets play a key role initiating normal haemostasis and pathological thrombosis. Several studies have shown that ECMO may lead to thrombocytopenia through platelet consumption [23, 24], though Abrams and colleagues found no relationship between ECMO duration and the occurrence of severe thrombocytopenia, suggesting that platelet counts might not fully reflect bleeding risk [25]. Consistent with the latter study, we found no significant change in platelet numbers. While platelet numbers were unaffected by changes in ECMO flow, studies continue to document the impact of high shear stress on platelet functionality, including impaired responses to ADP in adults [26] and children [27] on ECMO. In our study, ADP- and TRAP-induced platelet aggregation decreased over time with both high- and low-flow regimes, while RISTO-induced aggregation was reduced during low-flow only. Although our study failed to reveal any corresponding change in the number of Gp1bα or P-selectin receptors on platelets, plasma levels of P-selectin increased over time with both flow regimens. P-selectin belongs to the selectin family of cell adhesion molecules and supports the initial tethering of leukocytes to activated endothelial cells and platelets [28]. The combined loss of platelet reactivity, in the face of increased plasma P-selectin, suggests ECMO-induced platelet activation and fatigue. ECMO has been shown to increase P-selectin levels at 4 h in neonates, activating platelets whilst impairing their ability to aggregate [29].

Increased shear stress, as seen with ventricular assist devices and aortic stenosis, has been reported to cause alterations in the configuration of vWF and reduce the levels of HMW multimers [30]. The multimers of vWF uncoil under high shear stress, exposing their binding sites to the platelet receptor Gp1bα and, thus, stimulating activation [31]. Interestingly, our study revealed degradation of HMW vWF multimers with low-flow, while high-flow maintained similar levels of HMW and low-molecular-weight (LMW) multimers. These changes indicate that HMW vWF multimers are being cleaved into smaller fragments [32, 33] with low-flow. In our study, plasma levels of ADAMTS13, the vWF-cleaving protease, did not change over time. There is potentially less damage to HMW multimers with high flow, since contact time with the membrane is reduced. Blood is exposed to a large surface area with the Quadrox PLS-i (1.8 m^2^), and 1.5 L/min might not provide enough coverage, leading to areas of stagnation. We speculate that low pump flow leads to localised stasis and may precipitate more clot formation in the circuit oxygenator, as observed in computational modelling studies [34, 35].

Our thromboelastometry measurements of the intrinsic pathway demonstrated that low-flow prolonged the HEPTEM-CFT, associated with a corresponding decrease in MCF, consistent with thrombocytopenia/platelet dysfunction. This suggests that low-flow rate affects both the initiation and propagation phase of coagulation. With respect to the extrinsic pathway, PT was unchanged while EXTEM-MCF was reduced with both high- and low-flow, implying that changes to the extrinsic pathway may be independent of flow dynamics. This coagulation derangement may be a dilutional effect from fluid replacement.

Levels of the naturally occurring anticoagulant protein C were elevated with high-flow. This is consistent with studies in CPB that have demonstrated increased levels of activated protein C during and after bypass [36]. The detection of d-dimers, or fibrin degradation products generated by reactive fibrinolysis, with low flow, is indicative of coagulation activity. However, elevated d-dimer levels can also be seen with clinical conditions, such as inflammation, or generated by the foreign surfaces inherent with extracorporeal organ replacement therapies [37]. An increase in d-dimer concentration may, therefore, be a predictor of clot formation in the membrane oxygenator at low flow [37, 38].

Our study has a number of limitations. First, this was a study of short duration, which might not elucidate the longer-term impact of ECMO that occurs in the real-life clinical scenario. Second, we only assessed one type of oxygenator and two different flow rates (1.5 and 4 L/min), as the Quadrox PLS-i has a recommended blood flow rating of 0.5–7 L/min. Thirdly, the biological component, including any endothelial interaction, was excluded. Finally, the effect of cannula size on haemostatic changes was not considered, including the effect of shear stress induced by the edges of the venous cannulas. Future studies would focus on animal models of ECMO and assess multiple types of oxygenators at different flow rates.

## Conclusions

Current management of lower ECMO blood flows balances the perceived benefits of reduced shear stress to the blood elements, and therefore reduced haemolysis, with an increased risk of thrombosis. However, our ex-vivo model enabled us to demonstrate that low-flow (1.5 L/min) during the initial hours of ECMO leads to increased haemolysis, decreased platelet aggregation, and the loss of HMW multimers. These changes to haemostasis may be due to areas of stagnation or turbulence within the oxygenator, extending the contact time between the blood and the circuit’s artificial surface. Our results suggest that there might be an ideal minimum flow rate for each oxygenator, and that haemostatic changes could result from deviations outside the optimal range. Longer-duration studies are required to characterise ECMO-induced haemostatic changes beyond 6 h with different flow rates.

## Data Availability

The datasets generated during and/or analysed during the current study are available from the corresponding author on reasonable request.

## Abbreviations

ADP: Adenosine diphosphate
APC: Allophycocyanin
AT: Antithrombin
AUC: Area under the aggregation curve
BV: Brilliant violet
CFT: Clot formation time
CT: Clotting time
ECCO_2_R: Extracorporeal carbon dioxide removal
ECMO: Extracorporeal membrane oxygenation
FBE: Full blood examination
FITC: Fluorescein isothiocyanate
Hb: Haemoglobin
HMW: High molecular weight
LMW: Low molecular weight
MCF: Maximum clot firmness
PLT: Platelet
PT: Prothrombin time
RISTO: Risocetin
ROTEM: Rotational thromboelastometry
SAGM: Saline-adenine-glucose-mannitol
TRAP-6: Thrombin receptor activating peptide-6
VA: Venoarterial
VV: Venovenous
vWF: von Willebrand factor
WCC: White cell count
